# The poverty prevention effects of health insurance: evidence from China's basic medical insurance program

**DOI:** 10.3389/fpubh.2025.1576146

**Published:** 2025-04-24

**Authors:** Xinyue Yuan, Xujin Yang, Xianhua Zhou

**Affiliations:** ^1^School of Journalism, Communication University of China, Beijing, China; ^2^School of Mathematics and Statistics, Hunan University of Technology and Business, Changsha, Hunan, China; ^3^China Institute for Actuarial Science, School of Insurance, Central University of Finance and Economics, Beijing, China

**Keywords:** basic medical insurance, illness-induced poverty, moral hazard, preferential subsidies, poverty prevention effects

## Abstract

**Background:**

China's Basic Medical Insurance (BMI) system is designed to alleviate illness-induced poverty. This study aims to evaluate the effectiveness of BMI in pre-venting households from falling into poverty, uncover its underlying mechanisms, and propose a preferential subsidy policy to address existing inequities.

**Methods:**

Panel data from the 2010–2018 China Family Panel Studies (CFPS) were used. We employed Probit and Heckman models to assess how BMI enrollment influences poverty likelihood, focusing on rural–urban differences, total healthcare expenditures, and out-of-pocket (OOP) spending. We also proposed a tiered, progressively subsidized policy—“income segmentation with progressive reimbursement”—as a potential strategy to bolster poverty prevention among low-income households.

**Results:**

Our findings indicate that BMI enrollment significantly reduces the probability of house-holds becoming poor, with more pronounced effects in rural areas than in urban ones. Following enrollment, total healthcare expenditures rise substantially and health outcomes improve, yet OOP expenses do not show a corresponding decline and can even increase for urban residents. Moreover, higher-income groups benefit more from BMI, whereas low-income families face financial constraints that limit their ability to fully utilize these benefits.

**Conclusions:**

Despite BMI's success in improving health out-comes, moral hazard remains a concern, particularly in urban settings. A tiered, progressively subsidized approach can more effectively support low-income households, lowering their risk of falling back into poverty due to illness. These results underscore the importance of refining insurance policies to ensure that vulnerable populations can fully benefit from healthcare coverage.

## 1 Introduction

As one of the largest social security programs worldwide, China's Basic Medical Insurance system has gradually established a three-tier framework covering both urban and rural populations (Urban Employee Basic Medical Insurance, Urban Resident Basic Medical Insurance, and the New Rural Cooperative Medical System) since the early 21st century. Its core objective is to alleviate illness-induced poverty through risk-sharing mechanisms. By the end of 2023, the enrollment rate in China's BMI had stabilized at over 95%, achieving preliminary universal coverage (1). However, as the system enters a stage of high-quality development, the effectiveness of its poverty-reduction outcomes, the complexity of its mechanisms, and the equity of beneficiary groups have increasingly become focal points of academic debate. On the one hand, numerous studies confirm that health insurance mitigates financial risks by reducing Out-of-Pocket (OOP) healthcare expenses (2). On the other hand, moral hazard may lead to an escalation in OOP costs (3)^1^, which could offset its poverty-reduction dividends. Moreover, the urban–rural segmentation embedded in the system design and disparities among different income groups may exacerbate social inequality (4). Against this backdrop, clarifying the Poverty Prevention Effects of BMI, its underlying mechanisms, and group heterogeneity is critical for optimizing policy design and advancing the goal of common prosperity. It is worth noting that although China has made significant achievements in poverty alleviation and prevention through medical insurance, there are crucial differences between its health insurance model and those of other countries in poverty prevention. The advantages and disadvantages of different medical insurance models are presented in Table 1.

**Table 1:** Advantages and disadvantages of different health insurance models.

**Health insurance model**	**Representative countries**	**Advantages and disadvantages**
Chinese health insurance model	China	**Advantages:** the health insurance fund is reimbursed in different segments for government agencies, urban employees, and basic medical insurance for urban and rural residents, balancing the efficiency of “more contributions, more benefits” with fairness for low-income rural populations. **Disadvantages:** the reimbursement catalogs of the existing triple-tier health insurance system vary significantly, making it difficult to fully alleviate the medical cost burden for middle- and low-income groups.
National health insurance model	United Kingdom, Canada, Australia, Sweden	**Advantages:** the health insurance fund is primarily financed through taxation, ensuring stable funding and strong social pooling capacity. Public hospitals directly provide free services to residents, guaranteeing basic medical coverage for low-income groups and enhancing fairness. **Disadvantages:** the efficiency of public hospital health services is low. There is no well-established constraint mechanism between healthcare providers, consumers, and third-party payers, leading to a lack of cost awareness among both supply and demand sides and resulting in waste of healthcare resources. Additionally, health insurance fund expenditures are increasing, placing a growing financial burden on the government.
Commercial health insurance model	United States	**Advantages:** this model helps meet the diverse medical service needs of different population groups. Its financing channels are reliable, and the funding pool remains relatively stable. **Disadvantages:** patients have limited choices in seeking medical treatment. Healthcare consumption is primarily regulated by the market, lacking an effective supervision and control mechanism.
Social health insurance model	Germany, France, Netherlands	**Advantages:** each insured member contributes to economic redistribution, optimizing social welfare. The financing is jointly supported by individuals, enterprises, and the government, ensuring relatively high funding stability. **Disadvantages:** prior enrollment and premium payments are required to qualify for health insurance benefits, and the management cost of the insurance fund is relatively high.
Mandatory savings-based health insurance model	Singapore	**Advantages:** the coverage extends to a broad population, and the insurance fund is used efficiently. The MediShield and medical assistance programs provide additional “horizontal” aid for individuals with special needs, further enhancing fairness. **Disadvantages:** there is a lack of scientific and standardized guidance for individuals when utilizing insurance funds.

Existing studies on the poverty-reduction effects of health insurance primarily follow two main lines of inquiry. The first focuses on the direct impact of health insurance on households' financial vulnerability. Research indicates that insurance can lower Out-of-Pocket (OOP) expenditures and reduce the incidence of catastrophic health spending (5), though its effectiveness is moderated by the level of coverage, type of illness, and households' economic endowments (6–8). The second line of inquiry examines the long-term impacts of insurance on health status and human capital. Drawing on Grossman's health capital theory (9), many scholars argue that health insurance enhances access to medical services, thereby reinforcing incentives for health investment (10, 11), which in turn improves labor productivity (12, 13). However, due to information asymmetry in the healthcare market, the coordination of health insurance further exacerbates the issue of moral hazard. Moral hazard manifests as excessive medical consumption (14, 15), leading to the waste of medical resources (16, 17), thereby reducing overall social welfare (18–21).

It is noteworthy that the current literature has three main limitations. First, analyses of poverty-reduction mechanisms frequently concentrate on a single channel—reducing OOP expenditures—without a comprehensive examination of total medical costs, the burden of out-of-pocket payments, and health improvements. Second, discussion of heterogeneity across rural and urban households and among different income groups remains limited, particularly regarding systematic explanations of how “insurance dividends” might favor higher-income populations. Third, most policy de-sign studies rely on theoretical simulations and lack precise policy proposals grounded in micro-level behavioral data.

Building on data from the 2010–2018 China Family Panel Studies, this paper endeavors to extend existing research in three ways. First, at the theoretical level, it pro-poses a three-dimensional framework—encompassing “costs, burdens, and health”—to reveal the multiple mechanisms underpinning the Poverty Prevention Effects of health insurance, with particular attention to how moral hazard may erode these effects. Second, methodologically, it combines Probit and Heckman selection models to correct for endogeneity issues arising from self-selection (for instance, healthier individuals being more likely to enroll), thereby enhancing the robustness of the estimates. Third, at the policy level, drawing on findings from heterogeneity analyses, the paper puts for-ward a preferential subsidy design based on “income segmentation with progressive reimbursement,”^2^ offering a feasible solution to the dilemma of “easy enrollment but limited benefits” faced by low-income households.

The rest of this research is structured as follows. The next section presents the method. The third section provides the results and discussion. The conclusions and policy recommendations are offered in the last section.

## 2 Methods

### 2.1 Policy context and theoretical hypotheses

#### 2.1.1 Institutional background

The development of China's Basic Medical Insurance system has been closely intertwined with the country's poverty reduction efforts. In the 1990s, as China transitioned from a planned economy to a market-based one, the traditional publicly funded healthcare system began to dismantle. The coverage rate of the rural Cooperative Medical System plummeted from 90% in 1978 to below 10% in 2003. To address ill-ness-induced poverty, the government introduced the New Rural Cooperative Medical Scheme (NRCMS) in 2003 and piloted the Urban Resident Basic Medical Insurance (URBMI) in 2007. In 2016, these two programs were consolidated into the Urban–Rural Resident Basic Medical Insurance system, forming a nationwide coverage framework alongside the Urban Employee Basic Medical Insurance (UEBMI).

Nonetheless, notable structural contradictions persist under this broad-based coverage framework. First, the urban–rural dual structure results in uneven benefit levels. Historically, both NRCMS and URBMI have featured lower funding bench-marks and reimbursement rates compared to UEBMI. In 2018, the per capita funding for UEBMI was about four times that of the urban–rural resident program, and reimbursement rates for hospital stays in tertiary facilities differed by more than 20 percentage points (4). While rural residents generally have high enrollment rates, the weak service capacity of grassroots healthcare institutions limits the actual scope of reimbursement (5). Second, escalating medical costs coincide with persistent OOP burdens. Following the 2009 healthcare reform, expanded insurance coverage released a backlog of medical demand but also induced moral hazard on the supply side: hospitals offset cost-control pressures by ordering unnecessary tests or prescribing high-priced medications not covered by insurance. Finally, the distribution of insurance benefits shows a bias toward higher-income groups. Wealthier individuals are better positioned to utilize policies that offer higher reimbursement rates and superior services, whereas low-income households, constrained by limited financial resources, often must forgo essential medical care (7). Consequently, exploring preferential policy instruments to rectify systemic biases emerges as a key pathway for overcoming the “poverty prevention bottleneck.”

#### 2.1.2 Theoretical hypotheses

A growing body of evidence confirms that the unpredictability of medical expenses is a key driver of households falling into poverty (22, 23). From the perspective of development economics, medical insurance smooths households' healthcare expenditure fluctuations through risk-sharing mechanisms, a proposition that can be traced back theoretically to Townsend's risk-pooling model (24). Empirical studies show that China's New Rural Cooperative Medical Scheme reduced the incidence of catastrophic health spending among rural households by 12–18% (3), and that Medi-caid expansion in the United States lowered the poverty rate by 2.3 percentage points (18). Given China's typical urban–rural dual structure, differences in policy effectiveness between rural and urban areas also merit closer examination. Therefore, we propose the following hypothesis:

**Hypothesis 1:** Enrolling in medical insurance effectively reduces the probability that households will fall into poverty, with stronger effects in rural areas.

Classic theoretical debates persist regarding the leverage effect of health insurance on medical expenditures. According to Grossman's health demand model (9), price elasticity leads insured individuals to increase healthcare utilization—a claim supported by the RAND Health Insurance Experiment, which reported that every 10% decrease in cost-sharing raises total medical spending by 1.5–6.5% (25). More recent research suggests a complex, non-linear relationship in China: OOP costs for inpatient services may decrease, while outpatient expenses may increase (26), so overall changes in OOP require deeper scrutiny. Given the likelihood of higher total medical spending under insurance coverage, health outcomes are particularly important to consider. Studies on Medicare in the United States found that broader coverage improved health and reduced mortality among older adults by 8% (27), while also boosting labor supply (28, 29). Accordingly, we propose two further hypotheses:

**Hypothesis 2:** Enrolling in medical insurance increases total medical expenditures due to insurance's leverage effect, but because of moral hazard in healthcare services, Basic Medical Insurance does not necessarily reduce households' OOP spending.
**Hypothesis 3:** Enrolling in medical insurance significantly promotes healthcare utilization and thereby improves residents' health status.

A cross-country comparison in 21 nations found that high-income groups consistently enjoy higher-quality healthcare services—even in countries with universal coverage (30). Underlying mechanisms include higher-income individuals' stronger capacity to process health information, the unequal geographic accessibility of healthcare services, and the technical biases embedded in reimbursement catalogs (31). However, there are important counterexamples: Brazil's Unified Health System, with its strict referral procedures, increased outpatient visits by low-income groups 28% more than those by high-income groups (32). Such institutional heterogeneity indicates that the policy parameters of health insurance—such as deductibles and coverage lists—can reshape the distribution of benefits. Existing research has paid insufficient attention to the dynamic interplay between institutions and markets. Focusing on China's Basic Medical Insurance system and adopting an income-stratified perspective, this paper posits:

**Hypothesis 4:** The benefits of medical insurance differ by income level, with high-income groups reaping greater gains.

Feldstein's classic analysis demonstrated that when the demand for healthcare is relatively price-inelastic (33), differentiated compensation can achieve Pareto improvements. In practice, many developing countries adopt regressive deductibles and copayments, yet policy outcomes remain contested. Colombia's tiered subsidy system increased hospital admissions among poor households by 17% but did not significantly reduce their OOP burden (34). In particular, low-income individuals often cannot afford more expensive medications (35). Therefore, preferential policies that grant higher reimbursement rates to low-income groups may be more effective in improving social welfare. Hence, we propose:

**Hypothesis 5:** A tiered reimbursement policy should be adopted for different in-come groups, offering higher reimbursement rates for lower-income households.

### 2.2 Econometric model and data

#### 2.2.1 Data source and variable selection

This study utilizes five waves (2010, 2012, 2014, 2016, and 2018) of panel data from the China Family Panel Survey (CFPS), conducted by the Institute of Social Science Survey at Peking University. These data encompass the full implementation and notable achievements of China's Basic Medical Insurance (BMI) system, and include information on household income and expenditures, various insurance programs, health status, and educational attainment—factors directly relevant to this research.

Sample selection followed these criteria: Excluding “non-standard” households where the head of the household is younger than 25 or older than 60 years of age; Eliminating households composed only of older adults (≥65 years) or minors (<18 years); Performing listwise deletion of observations missing over 30% of key variables; Detecting and removing outliers for continuous variables (income, medical expenditures, etc.) using the Tukey fences method. After applying these filters, the final valid sample includes 29,526 observations. Table 2 details the logic used for variable selection and provides variable definitions.

**Table 2:** Variable definitions.

**Variables**	**Symbols**	**Definitions**
**Explained variables**		
Poverty dummy	*ispoor*	Whether the household is impoverished (1: poor household; 0: non-poor)
Household income	*income*	Per capita net income of the household
Total medical expenditure	*med*	Per capita total medical expenditure in the past year
Out-of-pocket Medical Costs	*medse*	Per capita out-of-pocket medical expenditure in the past year
**Core explanatory variable**		
Insurance enrollment	*ins*	Whether the household participates in basic health insurance (1: yes; 0: no)
**Control variables**		
Commercial insurance	*cins*	Whether the household purchased commercial insurance in the past year (1: yes; 0: no)
Policy context	*policy*	Whether survey year falls under the “Targeted Poverty Alleviation” policy framework (1: yes; 0: no)
Health status	*health*	Average household health status (1: Very unhealthy; 2: Unhealthy; 3: Fair; 4: Relatively healthy; 5: Healthy; 6: Very healthy)
Education attainment	*education*	Highest education level among adult members (0: Illiterate/semi-illiterate; 1: Below primary; 2: Primary; 3: Junior high/technical secondary; 4: Senior high; 5: College; 6: Undergraduate; 7: Master's; 8: Doctoral)
Older adult dependency ratio	*eld*	Proportion of older adult members in household
Child dependency ratio	*child*	Proportion of minor members in household
Household size	*size*	Number of household members
GDP per Capita	*gdp*	Local GDP per capita
Urban-rural division	*urb*	Household location (1: Urban; 0: Rural)

#### 2.2.2 Econometric model specification

##### 2.2.2.1 Probit model

When examining the binary causal relationship of “the poverty prevention effect of health insurance,” the choice of the Probit model is primarily based on the assumption of a normally distributed error term, as well as the traditional use of Probit regression in economics and health economics research. Compared to the Logit model, the estimation results of Probit and Logit are generally similar in direction and significance. However, in many cases, Probit is more convenient for integration with certain extended methods and aligns more intuitively with the normal distribution assumption when interpreting marginal effects. Additionally, the propensity score matching (PSM) method primarily focuses on eliminating intergroup differences at the observational level through matching. However, when there is potential endogeneity or unobservable individual heterogeneity, PSM may fail to fully identify the true causal effect. In contrast, using Probit regression combined with appropriate control variables allows for a better identification of the causal effect of “insurance participation” on “poverty status (0—non-poor household; 1—poor household),” making the Probit model more practical and interpretable. The model is specified as follows:


(1) $$\begin{array}{l} \mathrm{Pr}\left(ispoor_{i,\;t}=1\right)=\phi \left(c+\alpha •ins_{i,\;t}+X_{i,\;t}•+\varepsilon_{i,\;t}\right) \end{array}$$

Where *ins*_*i, t*_ denotes the poverty dummy variable, with subscript, *i* indicating the household and *t* indicating the year. *X*_*i, t*_ represents a set of control variables—including average household health status, average educational attainment of adults, and the old-age dependency ratio—while ε_*i, t*_ is the random error term.

##### 2.2.2.2 Heckman model

To further explore the mechanisms through which medical insurance prevents poverty in China and to address potential sample selection bias, this paper employs a Heckman two-step model. The rationale is that a household may or may not choose to seek medical care, and such a decision—being determined by the household itself—can introduce selection bias. The specific steps are as follows:


(2) $$\begin{array}{l} \ln\left(M_{i,\;t,\;k}|ispoor_{i,\;t}=1\right)=\theta_{0}+\theta_{1}•ins_{i,\;t}+X_{i,\;t}•\Theta +\xi_{i,\;t} \end{array}$$

Where *M*_*i, t*, 1_ refers to total medical expenditures, and *M*_*i, t*, 2_ refers to out-of-pocket medical expenses.

#### 2.2.3 Descriptive statistics

As shown in Table 3, the mean value of the household poverty indicator (*ispoor*) is 0.11 (standard deviation 0.32), indicating that approximately 11% of households in the sample are in poverty. There is notable heterogeneity between rural and urban areas. The mean log of per capita total medical expenditures (ln*_med*) is 8.08 (standard deviation 1.47), while the mean log of per capita out-of-pocket expenditures (ln_*medsec*) is 7.23 (standard deviation 1.68). These large standard deviations and wide ranges reflect a right-skewed distribution of medical spending and substantial individual variation in out-of-pocket risk.

**Table 3:** Descriptive statistics.

**Variables**	**Mean**	**Std.Dev**	**Min**	**Max**	***N***
*ispoor*	0.11	0.32	0.00	1.00	29,526
ln*_med*	8.08	1.47	1.61	13.84	29,526
ln*_medse*	7.23	1.68	0.00	13.14	29,526
ln*_income*	9.48	1.06	2.30	15.01	10,406
ln*_med_rmb*	5.50	3.54	0.00	13.84	10,406
*ins*	0.76	0.43	0.00	1.00	29,526
*cins*	0.29	0.45	0.00	1.00	29,526
*policy*	0.57	0.50	0.00	1.00	29,526
*heealth*	4.23	1.07	1.00	6.00	10,406
*education*	2.76	1.18	0.00	8.00	29,526
*eld*	0.10	0.18	0.00	1.00	29,526
*child*	0.14	0.14	0.00	0.67	29,526
*size*	4.11	1.81	1.00	21.00	29,526
ln*_gdp*	10.73	0.44	9.49	11.86	29,526

Household per capita income (ln_*income*) averages 9.48 (standard deviation 1.06), indicating a relatively concentrated distribution but with extreme values present. The coverage rate of Basic Medical Insurance (*ins*) is 76%, leaving 24% of households uninsured. By contrast, the coverage rate of commercial health insurance (*cins*) is only 29%, suggesting that the supplemental insurance market remains underdeveloped.

The dummy variable for the targeted poverty alleviation policy (*policy*) has a mean of 0.57, indicating that 57% of observations fall within the post-2015 period of policy implementation and reform. Households' self-rated health status (*health*) averages 4.23—close to a “relatively healthy” evaluation—though individual differences are pronounced (standard deviation 1.07). The mean education level (*education*) is 2.76, corresponding roughly to junior high school or secondary vocational education, suggesting potential constraints on human capital accumulation.

Regarding demographic structure, the old-age dependency ratio (*eld*) is 0.10, while the child dependency ratio (*child*) is 0.14. The mean of the logged provincial GDP (ln_*gdp*) is 10.73 (standard deviation 0.44), indicating relatively moderate interprovincial economic disparities. Overall, the data exhibit marked heterogeneity in medical burdens, income distribution, and insurance enrollment.

## 3 Results

### 3.1 Poverty prevention effects of China's basic medical insurance

Table 4 presents both the full-sample baseline results as well as subsamples for urban and rural households, facilitating a robustness check for our findings. As shown in Table 4, the coefficient of the insurance enrollment variable (ins) is −0.1814 and is statistically significant at the 1% level, indicating that purchasing Basic Medical Insurance (BMI) reduces the probability of falling into poverty. Notably, the reduction in poverty probability is more pronounced among rural households, suggesting that BMI enrollment is especially effective at mitigating illness-induced poverty in rural areas. This finding supports Hypothesis 1.

**Table 4:** Baseline regression of the poverty prevention effects of medical insurance.

**Variables**	**Full-sample**	**Urban-sample**	**Rural-sample**
*ins*	−0.181^***^ (0.026)	−0.114^***^ (0.038)	−0.238^***^ (0.037)
*cins*	−0.336^***^ (0.027)	−0.386^***^ (0.039)	−0.320^***^ (0.039)
*policy*	−0.325^***^ (0.022)	−0.203^***^ (0.034)	−0.412^***^ (0.030)
*education*	−0.298^***^ (0.011)	−0.332^***^ (0.016)	−0.292^***^ (0.018)
*eld*	0.168^***^ (0.060)	−0.162^*^ (0.093)	0.409^***^ (0.081)
*child*	0.093 (0.082)	−0.176 (0.123)	0.342^***^ (0.111)
*size*	0.063^***^ (0.006)	0.092^***^ (0.009)	0.049^***^ (0.008)
*ln_gdp*	−0.305^***^ (0.026)	−0.260^***^ (0.038)	−0.404^***^ (0.036)
constant	2.838^***^ (0.275)	2.386^***^ (0.417)	3.883^***^ (0.380)
*N*	29,526	14,660	14,866

^***^, ^**^, and ^*^ denote significance at the 1, 5, and 10% levels, respectively.

Regarding other control variables, the coefficient for commercial insurance enrollment is also significantly negative at the 1% level, implying that owning commercial insurance policies further decreases a household's likelihood of falling into poverty. The policy dummy variable—reflecting the implementation of “targeted poverty alleviation” measures—is significantly negative at the 1% level, indicating that after these measures took effect, BMI was even more successful in preventing households from becoming poor due to health-related shocks. Education level similarly shows a significant negative coefficient at the 1% level, highlighting that higher educational attainment corresponds to higher income and a lower probability of poverty.

By contrast, the old-age dependency ratio is significantly positive at the 1% level, suggesting that households with a higher proportion of older adult members face greater health risks and a higher probability of falling into poverty. However, for the urban subsample, the coefficient is significantly negative, which may be explained by the presence of higher pension incomes among urban older adult populations. Household size is positively associated with the probability of poverty at the 1% level, indicating that larger families tend to incur higher aggregate health risks. Finally, the logged GDP measure is significantly negative at the 1% level, suggesting that households in more economically developed regions are less likely to fall into poverty.

### 3.2 Robustness checks

To further assess the robustness of the baseline findings, two sets of additional tests were conducted: Excluding a portion of the sample: specifically, the top 20% of high-income households were removed; Subsample analysis by region: separate estimations for the eastern, central, western, and northeastern regions of China. The robustness check results are presented in Tables 5, 6. As shown by the findings, neither method alters the sign or significance of the results, indicating that the baseline estimates are robust.

**Table 5:** Robustness analysis: poverty prevention effects after excluding top 20% high-income households.

**Variables**	**Full-sample**	**Urban-sample**	**Rural-sample**
*ins*	−0.211^***^ (0.027)	−0.141^***^ (0.039)	−0.262^***^ (0.039)
*cins*	−0.286^***^ (0.028)	−0.350^***^ (0.040)	−0.243^***^ (0.041)
*policy*	−0.309^***^ (0.023)	−0.176^***^ (0.034)	−0.398^***^ (0.032)
*education*	−0.272^***^ (0.012)	−0.315^***^ (0.017)	−0.266^***^ (0.019)
*eld*	0.151^**^ (0.062)	−0.171^*^ (0.096)	0.359^***^ (0.084)
*child*	−0.073 (0.084)	−0.330^***^ (0.124)	0.154 (0.116)
*size*	0.044^***^ (0.006)	0.076^***^ (0.010)	0.026^***^ (0.008)
*ln_gdp*	−0.237^***^ (0.027)	−0.190^***^ (0.040)	−0.341^***^ (0.038)
constant	2.244^***^ (0.294)	1.738^***^ (0.443)	3.402^***^ (0.405)
*N*	29,526	11,729	11,893

^***^, ^**^, and ^*^ denote significance at the 1, 5, and 10% levels, respectively.

**Table 6:** Subsample robustness test: regional estimates of poverty prevention policies.

**Variables**	**Eastern China**	**Central China**	**Western China**	**Northeast China**
*ins*	−0.146^***^ (0.048)	−0.245^***^ (0.057)	−0.204^***^ (0.047)	−0.174^***^ (0.066)
*cins*	−0.323^***^ (0.051)	−0.366^***^ (0.055)	−0.351^***^ (0.046)	−0.232^***^ (0.083)
*policy*	−0.235^***^ (0.045)	−0.391^***^ (0.060)	−0.392^***^ (0.041)	−0.403^***^ (0.062)
*education*	−0.269^***^ (0.022)	−0.246^***^ (0.022)	−0.329^***^ (0.019)	−0.317^***^ (0.030)
*eld*	0.034 (0.120)	0.461^***^ (0.120)	0.111 (0.106)	0.165 (0.146)
*child*	−0.091 (0.162)	−0.052 (0.175)	0.395^***^ (0.133)	−0.153 (0.234)
*size*	0.062^***^ (0.011)	0.062^***^ (0.012)	0.066^***^ (0.010)	0.073^***^ (0.024)
*ln_gdp*	−0.606^***^ (0.063)	−0.121 (0.128)	−0.071 (0.065)	−0.221 (0.137)
constant	6.092^***^ (0.699)	−0.781 (1.344)	0.527 (0.665)	1.986 (1.511)
*N*	9,376	7,270	8,763	4,117

^***^, ^**^, and ^*^ denote significance at the 1, 5, and 10% levels, respectively.

### 3.3 Mechanism analysis of the poverty prevention effects of China's basic medical insurance

#### 3.3.1 Short-term effects of basic medical insurance

Although this study has revealed that purchasing basic health insurance can effectively reduce the likelihood of a household falling back into poverty due to catastrophic health expenses, further discussion is needed from the perspective of short-term effects regarding changes in total medical expenses and out-of-pocket medical costs after insurance enrollment. As shown in the second-stage results in Table 7, total medical expenses (med) significantly increase after enrolling in basic health insurance. In particular, the increase in medical expenses is more pronounced among rural residents. The reason is that rural residents generally have lower incomes, and the leverage effect of insurance encourages more of them to seek medical treatment. However, despite the significant increase in total medical expenses after purchasing basic health insurance, out-of-pocket medical costs show no significant change, and they even increase significantly among urban residents.

**Table 7:** Heckman model regression.

**Variables**	**Full-sample**		**Urban-sample**		**Rural-sample**	
	**Med**	**Medse**	**Med**	**Medse**	**Med**	**Medse**
**Second-stage regression results**						
*ins*	0.204^***^ (0.071)	0.140 (0.010)	0.202^*^ (0.105)	0.263^**^ (0.120)	0.256^***^ (0.093)	0.181 (0.132)
*cins*	−0.316^***^ (0.106)	−0.801^***^ (0.143)	−0.407^**^ (0.188)	−0.530^**^ (0.216)	−0.167 (0.129)	−0.638^***^ (0.175)
*child*	−0.844^***^ (0.236)	−1.083^***^ (0.323)	−0.984^***^ (0.323)	−1.672^***^ (0.371)	−0.862^***^ (0.315)	−0.735^*^ (0.438)
*education*	−0.352^***^ (0.066)	−0.787^***^ (0.090)	−0.358^***^ (0.131)	−0.459^***^ (0.151)	−0.313^***^ (0.072)	−0.804^***^ (0.101)
*size*	0.169^***^ (0.019)	0.180^***^ (0.027)	0.120^***^ (0.040)	0.152^***^ (0.046)	0.142^***^ (0.022)	0.136^***^ (0.030)
constant	5.787^***^ (0.286)	3.455^***^ (0.397)	6.001^***^ (0.554)	5.601^***^ (0.637)	6.067^***^ (0.292)	3.517^***^ (0.413)
**First-stage regression results**						
*ins*	−0.168^***^ (0.026)	−0.168^***^ (0.026)	−0.129^***^ (0.037)	−0.129^***^ (0.037)	−0.193^***^ (0.036)	−0.193^***^ (0.036)
*cins*	−0.354^***^ (0.027)	−0.354^***^ (0.027)	−0.397^***^ (0.038)	−0.397^***^ (0.038)	−0.342^***^ (0.039)	−0.342^***^ (0.039)
*education*	−0.297^***^ (0.011)	−0.297^***^ (0.011)	−0.324^***^ (0.015)	−0.324^***^ (0.016)	−0.306^***^ (0.017)	−0.306^***^ (0.017)
*child*	0.332^***^ (0.077)	0.332^***^ (0.077)	0.046 (0.116)	0.046 (0.116)	0.549^***^ (0.105)	0.549^***^ (0.105)
*size*	0.0622^***^ (0.006)	0.062^***^ (0.006)	0.093^***^ (0.009)	0.093^***^ (0.009)	0.047^***^ (0.008)	0.048^***^ (0.008)
*ln_gdp*	−0.402^***^ (0.026)	−0.402^***^ (0.026)	−0.314^***^ (0.039)	−0.314^***^ (0.039)	−0.549^***^ (0.036)	−0.549^***^ (0.036)
constant	3.688^***^ (0.280)	3.688^***^ (0.280)	2.803^***^ (0.432)	2.803^***^ (0.432)	5.244^***^ (0.383)	5.244^***^ (0.383)
Inverse-Mills ratio	1.415^***^ (0.221)	3.013^***^ (0.304)	1.311^***^ (0.447)	1.422^***^ (0.515)	1.183^***^ (0.212)	2.941^***^ (0.298)
*N*	29,526	29,526	14,660	14,660	14,866	14,867

^***^, ^**^, and ^*^ denote significance at the 1, 5, and 10% levels, respectively.

The reasons for this are, first, that outpatient expenses under China's health insurance system are not reimbursable, and medical costs for chronic diseases such as hypertension are not covered. Although government intervention has led to a further decline in drug prices, continuous medical expenses still result in a high financial burden. On the other hand, due to information asymmetry in the healthcare market, doctors, under the hospital revenue-generation system, may add unnecessary examinations and procedures, thereby increasing medical costs. As a result, overall out-of-pocket medical costs do not significantly decrease after participating in health insurance. Third, rural healthcare facilities are limited, and many rural residents, constrained by economic conditions and more frugal by nature, tend to “endure minor illnesses and seek hospitalization only for severe conditions.” In contrast, urban areas have better healthcare facilities, and urban residents visit doctors for minor illnesses more frequently. Fourth, China's basic health insurance system is divided into government agencies, urban employees, and urban-rural basic health insurance, with the latter combining the New Rural Cooperative Medical Insurance and Urban Resident Medical Insurance. Clearly, urban health insurance coverage is much higher than that in rural areas, and the leverage effect of urban insurance is stronger. This results in an insignificant increase in out-of-pocket medical costs for rural residents after enrolling in basic health insurance, whereas urban residents experience a significant increase. This indicates that moral hazard in healthcare is relatively pronounced. Clearly, these findings support Hypothesis 2.

#### 3.3.2 Long-term effects of basic medical insurance

We have shown that insured households incur significantly higher medical expenditures, implying that insurance coverage encourages more frequent healthcare utilization. Nevertheless, it is also crucial to assess whether this leads to improved health outcomes. As indicated in Table 8, following enrollment in Basic Medical Insurance (BMI), healthcare utilization rises, and residents' health status improves significantly. Notably, this improvement is especially pronounced among rural households. Because rural families typically face a heavier relative burden of healthcare costs, access to insurance leverages their out-of-pocket payments to secure a greater volume of medical services. These findings strongly validate Hypothesis 3.

**Table 8:** The impact of health insurance on residents' health status.

**Variables**	**Full-sample**	**Urban-sample**	**Rural-sample**
*ins*	0.139^***^ (0.027)	0.095^***^ (0.033)	0.200^***^ (0.045)
*eld*	−0.657^***^ (0.053)	−0.681^***^ (0.071)	−0.606^***^ (0.079)
*child*	1.976^***^ (0.084)	1.851^***^ (0.115)	2.162^***^ (0.123)
*education*	0.060^***^ (0.009)	0.036^***^ (0.011)	0.094^***^ (0.015)
*size*	0.012^**^ (0.005)	−0.0004 (0.008)	0.019^***^ (0.007)
*ln_gdp*	0.133^***^ (0.022)	0.072^**^ (0.030)	0.188^***^ (0.034)
*med_rmb*	−0.027^***^ (0.003)	−0.022^***^ (0.004)	−0.030^***^ (0.004)
constant	2.379^***^ (0.236)	3.179^***^ (0.326)	1.609^***^ (0.357)
*N*	14,096	6,870	7,226

^***^, ^**^, and ^*^ denote significance at the 1, 5, and 10% levels, respectively.

### 3.4 Further analysis: measures to prevent large-scale illness-induced poverty

#### 3.4.1 The poverty prevention effect of basic medical insurance across different income groups

To investigate the poverty prevention role of BMI, this study constructs a variable reflecting enrollment in BMI along with receipt of medical reimbursements—specifically, the interaction term of insurance purchase and reimbursement ratio (ins^*^hos_rmb). By examining how reimbursed households affect overall household income, we can evaluate the poverty prevention effectiveness of BMI.

As shown in Table 9, columns 1 to 5 represent five income groups ranked from lowest to highest, each accounting for 20% of the total population. The interaction terms in columns 1 to 4 are all insignificant, while only the fifth group is significant with a positive coefficient. This indicates that purchasing health insurance does not have a significant poverty prevention effect for middle- and low-income groups but exhibits a significant positive impact for high-income individuals. The reason is that high-income groups generally have better access to healthcare, allowing them to receive medical treatment quickly in areas with high-quality healthcare resources. They can also reduce out-of-pocket expenses through commercial supplemental insurance. Furthermore, high-income households tend to have a higher level of health literacy, making them more knowledgeable and adept at utilizing health insurance policies and reimbursement procedures, thereby maximizing their economic benefits across all stages of disease prevention and treatment. In contrast, middle- and low-income groups face constraints such as scarce medical resources, high out-of-pocket costs even after reimbursement, and a lack of familiarity with policy details and healthcare procedures. As a result, they struggle to achieve the same poverty prevention benefits despite having health insurance. Clearly, these findings strongly support Hypothesis 4.

**Table 9:** The impact of basic medical insurance on household income across different income groups.

**Variables**	**Full-sample**	**1**	**2**	**3**	**4**	**5**
ins^*^hos_rmb	0.009^**^ (0.004)	0.007 (0.009)	−0.004 (0.008)	0.005 (0.007)	−0.004 (0.007)	0.035^***^ (0.007)
*cins*	0.312^***^ (0.030)	0.352^***^ (0.090)	0.062 (0.063)	0.083 (0.060)	0.226^***^ (0.054)	0.214^***^ (0.056)
*policy*	0.181^***^ (0.027)	−0.008 (0.067)	0.161^***^ (0.058)	0.105^*^ (0.055)	0.152^***^ (0.052)	0.231^***^ (0.054)
*education*	0.297^***^ (0.014)	0.250^***^ (0.040)	0.241^***^ (0.035)	0.152^***^ (0.031)	0.192^***^ (0.028)	0.190^***^ (0.027)
*eld*	0.304^***^ (0.083)	0.288 (0.188)	−0.081 (0.200)	−0.030 (0.171)	0.511^***^ (0.167)	0.402^***^ (0.149)
*child*	−0.366^***^ (0.115)	−0.350 (0.309)	−0.155 (0.254)	−0.133 (0.240)	−0.060 (0.215)	−0.279 (0.215)
*size*	−0.071^***^ (0.008)	−0.071^***^ (0.018)	−0.016 (0.016)	−0.026 (0.016)	−0.070^***^ (0.017)	−0.023 (0.018)
*ln_gdp*	0.518^***^ (0.037)	0.330^***^ (0.117)	0.344^***^ (0.103)	0.115 (0.089)	0.364^***^ (0.071)	0.330^***^ (0.060)
constant	3.313^***^ (0.393)	5.114^***^ (1.237)	5.004^***^ (1.107)	7.919^***^ (0.954)	5.405^***^ (0.777)	5.747^***^ (0.669)
*N*	4,171	834	834	834	834	835

^***^, ^**^, and ^*^ denote significance at the 1, 5, and 10% levels, respectively.

#### 3.4.2 Evaluating a tiered design of China's basic medical insurance

Since the poverty prevention effects of Basic Medical Insurance (BMI) vary across income groups and prove less effective for households at lower income levels, this study proposes a preferential subsidy policy. Under this policy, households are grouped by income (from high to low) to adjust both total and out-of-pocket (OOP) costs for major illnesses. By increasing subsidies for low-income groups, the goal is to better protect them from poverty.

##### 3.4.2.1 Design of the hospitalization reimbursement subsidy

This approach adjusts re-imbursement rates by modifying both inpatient OOP expenses and total inpatient expenditures. Specifically, for each household in groups 1–5 (classified by per capita net income), the following transformations apply:


$$\begin{array}{r} hos_{-}all*=hos_{-}all\times k_{i},\;k_{i}=1.5-0.1\times i\\ hos_{-}\mathrm{self}*=hos_{-}\mathrm{self}\times l_{i},l_{i}=0.5+0.1\times i,i=1,2,\ldots ,5\\ hos_{-}rmb*=hos_{-}all*-hos_{-}self* \end{array}$$

Where *hos*_−_*self* and *hos*_−_*all* denote the household's initial inpatient OOP and total expenditures, respectively. $hos_{-}all^{*}$ and $hos_{-}self^{*}$ represent the adjusted figures under the proposed policy, while *k*_*i*_ and *l*_*i*_ are subsidy coefficients, and *i* = 1, 2, …, 5 denotes the five-tier income stratification. From these adjustments, we can derive a revised reimbursement rate for inpatient care.

Based on this tiered adjustment, each group's inpatient OOP and total expenditures are recalculated to obtain a new reimbursement ratio. This new ratio is then interacted with relevant model variables to generate a simulated value for household net income, which is subsequently regressed on the variables reflecting our tiered inpatient insurance subsidy policy. The results are shown in Table 10.

**Table 10:** Effects of adjusted inpatient reimbursement rates on household across different income groups.

**Variables**	**Full-sample**	**1**	**2**	**3**	**4**	**5**
ins^*^hos_rmb^*^	0.039^***^ (0.003)	0.081^***^ (0.008)	0.054^***^ (0.007)	0.046^***^ (0.008)	0.014^**^ (0.007)	0.033^***^ (0.007)
*cins*	0.224^***^ (0.026)	0.204^***^ (0.075)	0.039 (0.057)	0.043 (0.053)	0.210^***^ (0.052)	0.215^***^ (0.056)
*policy*	0.176^***^ (0.023)	0.081 (0.055)	0.132^***^ (0.051)	0.118^**^ (0.050)	0.153^***^ (0.049)	0.231^***^ (0.054)
*education*	0.234^***^ (0.013)	0.143^***^ (0.035)	0.187^***^ (0.030)	0.150^***^ (0.028)	0.182^***^ (0.027)	0.190^***^ (0.027)
*eld*	0.184^***^ (0.071)	−0.187 (0.152)	−0.104 (0.165)	−0.023 (0.155)	0.470^***^ (0.147)	0.408^***^ (0.149)
*child*	−0.308^***^ (0.102)	−0.623^**^ (0.278)	−0.119 (0.212)	0.019 (0.221)	−0.010 (0.205)	−0.294 (0.214)
*size*	−0.055^***^ (0.007)	−0.063^***^ (0.015)	−0.030^**^ (0.014)	−0.019 (0.015)	−0.061^***^ (0.015)	−0.022 (0.018)
*ln_gdp*	0.440^***^ (0.033)	0.327^***^ (0.100)	0.395^***^ (0.088)	0.154^*^ (0.079)	0.363^***^ (0.066)	0.329^***^ (0.060)
constant	4.325^***^ (0.351)	5.458 ^***^ (1.05)	4.648^***^ (0.947)	7.390^***^ (0.844)	5.417^***^ (0.726)	5.765^***^ (0.669)
*N*	4,171	834	834	834	834	835

^***^, ^**^, and ^*^ denote significance at the 1, 5, and 10% levels, respectively.

Comparing the outcomes before and after the reimbursement adjustments reveals the following: prior to the adjustment, the overall coefficient is significant, but Groups 1–4 are insignificant, and only Group 5 is significant. After implementing the proposed policy to adjust inpatient reimbursement rates and recomputing the simulated house-hold per capita net income in the model, the key explanatory variable remains significant in the full sample and is now also significant across all five income groups. This pattern indicates that the proposed policy enhancements can more effectively optimize the internal structure of medical insurance and improve the efficiency of poverty prevention. Clearly, this finding strongly supports Hypothesis 5.

## 4 Discussion

Based on the results from the previous section, this study reveals that the rural–urban disparity effect has significant policy implications. Health improvements are more pronounced for rural households after enrolling in Basic Medical Insurance (BMI), likely due to their initially lower healthcare accessibility, resulting in greater marginal benefits. On the other hand, the increased burden faced by urban households exposes the current system's inadequacy in addressing the “price elasticity trap”—urban residents are more likely to access high-cost medical services, and when non-reimbursed items are included in the reimbursement catalog, their actual out-of-pocket (OOP) expenses can rise with escalating treatment (4). This finding challenges the traditional view of measuring BMI effectiveness solely by coverage rates and underscores the need for a “quality-cost” dynamic balance mechanism. Additionally, the observation that higher-income groups benefit more confirms the “Matthew effect in healthcare resource allocation:” economically advantaged groups, with better access to information and bargaining power, are more adept at optimizing their medical consumption using re-imbursement policies (7). Meanwhile, low-income households, constrained by pre-payment capacity and opportunity costs for medical care, struggle to fully leverage the potential of insurance (6). This “hidden exclusion under a universal system” suggests that the current BMI system, segmented by identity (urban/rural or occupational), may exacerbate health inequalities across income classes.

This study provides three key insights for policy design: First, moral hazard control should be a key focus of healthcare reform. International experiences, such as the “gatekeeper system,” could be adopted to limit over-treatment through primary care referrals and tiered healthcare (16), alongside improving medical price negotiation mechanisms to curb supplier-induced demand. Second, to address the “easy to enroll, hard to benefit” dilemma faced by low-income groups, the “income segmentation with progressive reimbursement” subsidy scheme proposed here is practically feasible. By linking reimbursement rates to disposable household income (e.g., increasing the re-imbursement rate by 15–20 percentage points for the lowest 20% income group), this policy can achieve precise poverty reduction without increasing the overall fiscal bur-den. Finally, there is a need to strengthen the synergy between the BMI system and other social policies. For example, a medical cost exemption mechanism could be introduced to provide secondary compensation for the out-of-pocket expenses of low-income urban enrollees. Additionally, linking medical assistance with BMI benefits could create a multi-level poverty prevention safety net.

However, this study has several limitations: first, the timeliness of the data is a concern as major healthcare reforms, including the integration of urban and rural resident health insurance and drug procurement policies, took place after 2018. The effects of these policy changes have not been fully captured. Second, in the mechanism analysis, while Heckman models corrected for self-selection bias, the depiction of supply-side behaviors by healthcare providers remains inadequate. Future research could incorporate hospital-level data to quantify the extent to which supplier-side moral hazard in-fluences household medical expenditures. Third, the design of the preferential subsidy scheme needs to account for regional heterogeneity. For example, underdeveloped regions in central and western China may face fiscal sustainability challenges, which re-quires further cost-benefit simulation analysis.

From a broader theoretical perspective, this study offers a new dimension for understanding the poverty-reduction effects of social security systems. Traditional re-search has often focused on the direct effects of financial risk-sharing (2). This study, however, demonstrates that health human capital accumulation is a hidden pathway for BMI to prevent poverty. This “health-poverty” interaction mechanism suggests that when evaluating the effectiveness of healthcare policies, a dynamic cross-temporal analytical framework is necessary, particularly to assess the role of health improvement in increasing labor force participation and long-term household income (12). For developing countries currently expanding healthcare coverage, the study's conclusions carry important warnings: universal coverage is merely the first step in institutional development. The next phase of reform will focus on balancing “poverty prevention” with “cost control” through refined management.

## 5 Conclusions

Drawing on microdata from the 2010–2018 CFPS, this paper uses Probit and Heckman models to examine the poverty prevention effects and mechanisms of China's BMI, and further proposes a preferential subsidy policy to explore measures for pre-venting illness-induced poverty.

The findings indicate that BMI enrollment significantly reduces the probability of households falling into poverty, effectively mitigating illness-induced financial risks. Moreover, its impact is notably stronger among rural residents. Mechanistically, although total medical expenses rise sharply for individuals who join BMI, OOP costs do not show a significant overall change; in fact, OOP expenditures increase significantly among urban enrollees. At the same time, BMI substantially improves health out-comes—especially for rural residents—suggesting that, while enrolling in BMI pro-motes healthcare utilization and better health, moral hazard does exist, particularly in urban areas. This also helps explain why the poverty-prevention effect is more pronounced in rural communities. Further analysis reveals that higher-income groups benefit more from BMI, underscoring the need for targeted subsidy policies to safeguard low-income households against illness-induced poverty.

Therefore, this paper proposes the following recommendations. First, address the structural issue of “easy enrollment but difficult benefit utilization.” Implement a “one-stop” immediate settlement system to reduce upfront payments by patients. Expand outpatient reimbursement coverage and increase the reimbursement ratio for common and chronic illnesses. Strengthen the promotion of medical insurance policies to reduce difficulties caused by information asymmetry in reimbursements. Second, design a precise and sustainable fiscal subsidy mechanism, where the central government provides increased fiscal support to economically underdeveloped regions and disadvantaged groups, establishing a stable special transfer payment system. Local governments are responsible for matching local medical insurance funds, thereby establishing a clear division of responsibilities. Implement differentiated fiscal subsidies based on residents' income and payment capabilities. Third, implement differentiated medical insurance reimbursement to enhance benefits for low-income groups. For low-income and borderline poor groups, reduce reimbursement deductibles, increase reimbursement rates, and introduce a capped medical expense system to ensure medical expenditures for impoverished households do not exceed a certain proportion of their income, drawing on Germany's annual medical expense cap policy. Fourth, strengthen coordination mechanisms with central government support and local government implementation. Incorporate the effectiveness of medical insurance poverty alleviation into local performance evaluation systems, offer financial incentives to regions with strong implementation outcomes, and strictly hold accountable those with poor performance or misuse of funds to strengthen local motivation.

## Data Availability

The original contributions presented in the study are included in the article/supplementary material, further inquiries can be directed to the corresponding author.

## Footnotes

^1^The moral hazard referred to in this study indicates that, compared to residents who do not participate in basic health insurance, reimbursement under basic health insurance increases total medical expenses, yet out-of-pocket expenses do not decrease. The mismatch between reimbursement and medical expenses resulting from this phenomenon is what we define as moral hazard.

^2^“Income stratification and progressive proportion” refers to a policy in which income is categorized into different tiers from low to high, and as income increases, the proportion of out-of-pocket medical expenses gradually rises while the reimbursement rate correspondingly decreases.
